# Appeasing Pheromones against Bovine Respiratory Complex and Modulation of Immune Transcript Expressions

**DOI:** 10.3390/ani11061545

**Published:** 2021-05-25

**Authors:** Caroline Hervet, Justine Boullier, Marlène Guiadeur, Léa Michel, Laure Brun-Lafleur, Anne Aupiais, Jianzhong Zhu, Béatrice Mounaix, François Meurens, Fanny Renois, Sébastien Assié

**Affiliations:** 1 BIOEPAR, INRAE, Oniris, 44307 Nantes, France; caroline.hervet@inrae.fr (C.H.); justine.boullier19@hotmail.fr (J.B.); fanny.renois-meurens@oniris-nantes.fr (F.R.); sebastien.assie@oniris-nantes.fr (S.A.); 2 Institut de l’Élevage, 14310 Villers-Bocage, France; Marlene.guiadeur@idele.fr; 3 TERRENA Innovation, La Noëlle, 20199 Ancenis, France; lmichel@terrena.fr; 4 Institut de l’Élevage, 35652 Le Rheu, France; laure.brun-lafleur@idele.fr (L.B.-L.); anne.aupiais@idele.fr (A.A.); beatrice.mounaix@idele.fr (B.M.); 5 College of Veterinary Medicine, Comparative Medicine Research Institute, Yangzhou University, Yangzhou 225009, China; jzzhu@yzu.edu.cn; 6 Joint International Research Laboratory of Agriculture and Agri-Product Safety, Yangzhou 225009, China; 7 Department of Veterinary Microbiology and Immunology, Western College of Veterinary Medicine, Saskatoon, SK S7N5E3, Canada

**Keywords:** appeasing pheromone, bovine, respiratory infections, immune response, average daily gain

## Abstract

**Simple Summary:**

Bovine respiratory complex is still a major issue in bovine feedlots. Most of the time, antimicrobial molecules are used to manage these diseases with deleterious consequences for microbiota and the emergence and shedding of resistant bacteria. To improve bovine health and reduce the risk for cattle to develop respiratory infections, alternative molecules such as appeasing pheromones have been developed. In this study, we tested bovine appeasing pheromones in young bulls. We treated them at the beginning of the fattening period and measured zootechnical and health parameters over several weeks. We identified dual effects of pheromone treatment. Indeed, more respiratory clinical signs were observed in bulls who received the pheromone treatment on Day 8 than in bulls who did not, while it was the opposite on Day 30. Regarding the potential mechanism to explain the effect of the pheromone treatment, we identified an increased expression of transcripts associated with the expression of immune molecules involved in the recruitment of cells important to manage pathogens. Our study suggests a positive final effect of appeasing pheromones and opens the doors for future studies in beef cattle.

**Abstract:**

Bovine respiratory disease is still a major concern and has major economic impact. Another consequence of respiratory infections is the use of antimicrobial molecules to control bacterial pathogens. This can participate in the emergence and shedding of antimicrobial resistance that can threaten animal as well as human health. Appeasing pheromones with their capacity to reduce stress and thus their ability to preserve the functions of the immune system have been proposed to reduce the use of antimicrobial substances. In this study, we assessed the effect of appeasing pheromone administration on bovine health and performance during the fattening period. Zootechnical and health parameters and whole blood immune transcript expressions were measured over four weeks in bulls to determine the effect of the pheromone. We observed increased clinical signs on Day 8 (D8) and decreased clinical signs on D30 in bulls who received the pheromone and a higher expression of interleukin 8 transcripts in this group than in the control group on D8. Our results are overall in line with previous reports in livestock species. Further studies are needed to shed more light on the effect of appeasing pheromones and decipher their exact mechanisms of action.

## 1. Introduction

Bovine respiratory disease (BRD) complex remains a major cause of morbidity and mortality in bovine feedlots with major economic consequences [1]. Multiple pathogens, including various bacteria and viruses, and several host and environment factors such as air quality are involved in the pathogenesis of BRD complex [2,3,4,5]. To control bacterial infections, antimicrobial molecules are commonly used before (methaphylaxis) or after the detection of various clinical signs. These antibacterial treatments have major consequences not only on bovine respiratory and gut microbiota and pathogens but also on the shedding of resistant bacteria and resistance determinants into the surrounding environment [6,7,8]. This constitutes a serious health issue for animals and humans. Among the multiple factors involved in the pathogenesis of BRD, the stress has a determinant role [5,9]. It has been shown that stress is increased during the fattening period of young bulls for several reasons, including a recent weaning and the transportation of young bulls and their mixing in a new confined environment when they are coming from different farms [5,10,11,12,13,14]. Within a few days, multiple periods of stress show an impact on immune defense mechanisms of young bovine with consequences on the onset and persistence of respiratory infectious disorders [9,15,16,17].

To prevent the onset of respiratory infections during the fattening period of young bulls, vaccinations targeting the main pathogens can also be used [2,18]. Currently, vaccination is carried out when the young are gathering. However, this is quite late to enable a good protection at the beginning of the fattening period. New breeding management practices to limit the impact of stress on the immune system of young bovines (batches of bulls coming from a same farm, reduced transport time, etc.) could be considered. However, the implementation of these new practices could be challenging for the stakeholders. An alternative to reduce the impact of the stress could be the use in young animals of appeasing pheromones, as demonstrated in several species [19,20,21]. The production of these hormones during nursing exists in all mammals including bovine. In cows, there is a specific area between the two mammary chains [22]. Appeasing pheromones are produced by sebaceous glands of the sulcus. Synthetic analogs of the bovine appeasing hormone have been developed based on a mixture of fatty acids. They are similar to the pheromone produced by the dam at calving [23].

To the best of our knowledge, the effect of appeasing pheromone on bovine performance and disease incidence has been assessed in a limited number of studies [22,24,25,26]. Positive effects including increased performance and improved early detection of BRD signs were reported in dairy cattle as well as beef cattle.

Thus, in a global context of antimicrobial molecules use reduction, we decided to assess the effect of bovine appeasing pheromone on stress, the development of respiratory disorders, and whole blood immune transcript expression in young bulls over the four first weeks of fattening. Interesting observations were collected, showing an effect of the pheromones on bovine health and thus contributing to a better knowledge of this alternative tool.

## 2. Materials and Methods

### 2.1. Study Facilities

The study was carried out in four young bull fattening units in western France. The basic husbandry management of these fattening units is representative of standard management used in this area. In western France, 5–10-month-old young bulls are weaned and immediately transported to a sorting facility to be sorted by breed and body weight, forming new batches that fulfill the orders of the fatteners. The newly formed batches were transported to the fattening units for the entire fattening period. In the different fattening units, bulls from different suckler farms were mixed according to regular French farm practices. During the fattening period, young bulls were reared in barns composed of pens of 5–20 animals for a space allowance of 3.5–5.5 m^2^/bull. Young bulls were commonly fed with a complete diet composed of corn or grass silage and a mixture of cereals and urea.

### 2.2. Animals

The 265 animals utilized in the study were young Charolais bulls aged 317 days (d) (±51.8 d) at arrival at the sorting facility between January and March 2018 (Table 1). The animals came from multiple suckler farms. On arrival at the sorting facility, they were moved through a chute for a series of treatments. They were treated with doramectin anthelmintic (Dectomax, Elanco, Sèvres, France), vaccinated once with a vaccine containing modified-live bovine viral diarrhea virus (BVDV) and bovine respiratory syncytial virus (BRSV) (Rispoval RS-BVD, Pfizer, Paris, France), and then weighed. The average weight of the 265 animals was 366.7 kg (±30.8 kg). The farm bulls were housed and maintained in compliance with the French Ministry of Agriculture and Fishing standards for the protection of animals.

### 2.3. Batches Constitution and Pheromone Administration

Animals were sorted by weight, conformation, and farms of origin to form 23 homogeneous batches of 5–15 animals (Table 1). Two treatment groups were then randomly constituted. The pheromone group consisted of 159 young bulls in 14 batches and the control group consisted of 106 young bulls in 9 batches. For a given fattening unit, batches of pheromone and control group had comparable mean weight and included young bulls with similar conformations and from the same farms of origin.

### 2.4. Pheromone Administration

Before leaving the sorting facility, animals were moved again through a chute. Five milliliters of SecureCattle^®^ (Signs—Irsea Group, Apt, France) (pheromone group) or 5 mL of transcutol (Diethylene glycol monoethyl ether—Gattefossé, Saint-Priest, France) (control group), a high-purity solvent and powerful solubilizer associated with skin penetration enhancement in topical dosage forms, were deposited on the coat in the middle of the forehead—at the crossing point of two imaginary lines drawn between the eyes and the center of the base of the opposite horns. An Injecmaster^®^ automatic syringe without any needle was used to perform the pour-on administration (Génia, St. Hilaire de Chaléons, France). From there, contacts between young bulls from pheromone and control groups were prevented using an empty pen between groups in some fattening units and double fences with empty spaces between fences in the other groups.

### 2.5. Clinical Assessments

On Day 8 (D8) and D30, a veterinarian performed a visual examination for detection of BRD clinical signs (increase in respiratory rate, dyspnea, cough, nasal or ocular discharge, and depression). A diagnosis of BRD was established when the animal displayed: (i) nasal or ocular discharge or cough; and (ii) an increase in respiratory rate or depression. No clinical assessment was performed on D0 since BRD usually develop after the arrival at the fattening unit.

### 2.6. Activity and Behavioral Observations

Two observers blindly recorded activities, behaviors, and stereotypies (Table 2) on D0, D8, and D30 during a 6 h observation period. Both observers looked at the pheromone and placebo young bulls. They used instantaneous sampling of individual young bulls in each group using 5 min scan sampling. Data were recorded 48 times per day each, during 61 h observation periods from 9 a.m. to noon and from 2 p.m. to 5 p.m.

### 2.7. Zootechnical Performances

The duration of the fattening period was recorded for each young bull. Each young bull was weighed at the end of the fattening period. An individual average daily gain (ADG) (kg/d) for the entire fattening period was calculated as the change in body weight measured at the sorting facility and at the end of the fattening period divided by the number of days elapsed.

### 2.8. Immune Gene Expression Analysis Using Reverse-Transcription Quantitative Polymerase Chain Reaction

Total RNA was extracted from the blood of 15 (control) and 18 (pheromone) bulls randomly selected from each group on D0, after pheromone administration, and D8 using PAXgene Blood RNA Kit (Qiagen Courtaboeuf, France) as described by the manufacturer. RNA concentration was determined by measuring OD at 260 nm (OD260) and the RNA quality was assessed by calculating OD260/OD280 ratio NanoDrop spectrophotometer (NanoDrop Technologies, Wilmington, DE, USA). cDNA was generated with iScript Reverse Transcription Supermix for reverse-transcription quantitative polymerase chain reaction (RT-qPCR) (Bio-Rad, Hercules, CA, USA) from 400 ng of RNA free of genomic DNA per reaction. Diluted cDNA (2-fold) was combined with primer/probe sets and IQ SYBR Green Supermix (Bio-Rad) according to the manufacturer’s recommendations. The qPCR conditions, for the various genes tested in the study, were 95 °C for 30 s, followed by 40 cycles with denaturation at 95 °C for 5 s and annealing/elongation for 30 s at selected temperature—depending on the gene assessed. The efficiencies of all the qPCR assays were between 90% and 100%, and we carefully followed MIQE guidelines recommendations [27]. The sequences of the primers used in the study were published previously (see Table 3) [28,29,30,31,32,33]. Real-time assays were run on a Bio-Rad CFX96 (Bio-Rad). The specificity of the qPCR reactions was assessed by analyzing the melting curves of the products and verifying the amplicon sizes. Samples were normalized internally by simultaneously using the average Cycle quantification (*Cq*) of two suitable reference genes (Beta actin (ACTB) and Glyceraldehyde-3-phosphate dehydrogenase (GAPDH)) in each sample to avoid any artifacts of variation in the target gene [28]. Then, qPCR data were expressed as relative values after Genex macro analysis (Bio-Rad) [34] using the *Cq* from the samples for the different transcripts.

### 2.9. Statistical Analysis

Most calculations and statistical analyses were performed in the open-source environment R version 3.5.1. (R Development Core Team, Vienna, Austria). The characteristics of young bulls assigned to the two treatment groups were compared to assess homogeneity: Student’s *t*-test was used for continuous variables after checking normality using Shapiro–Wilk test. Then, a mixed effects logistic regression model using the glm function from the package lmertest was used to characterize the association between the exposure to pheromone and BRD diagnosis (diseased or healthy). The individual bull was the experimental unit. Fattening operation and pen were considered as random factors. An interaction between the date of clinical examination and BRD diagnosis was included in the statistical model.

Proportions and count were used to describe activity and behavioral observations. Because they displayed a skewed distribution, permutation tests with 1000 replications were used to calculate *p*-values. Hypothesis H0 is that an activity or behavioral observation has a significant effect. A mixed linear model was used with the initial dataset to test this effect, and the Chi-square statistic was calculated for each activity or behavioral observation. Then, 1000 replications were performed with a random permutation of group (pheromone or control) for young bulls at each replication while keeping the same proportion of pheromone groups and control groups, a random permutation of date of clinical examination (D0, D8, or D30), and a random permutation of number of young bulls per pen while keeping the same number of young bulls receiving pheromone as in the initial dataset. For each activity or behavioral observation, 1000 values of Chi-square statistic were then obtained. The 95% percentile of repartition of these 1000 values was calculated and compared with the value obtained with the initial dataset: if superior, then H0 is rejected, thus the activity and behavioral observation has a significant effect.

A mixed linear regression model using the lme function from the nlme package was used to characterize the association between the exposure to pheromone and ADG for the entire fattening period. The individual bull was the experimental unit. A “pen within fattening operation” nested random effect was added to the model.

Then, relative expressions of transcripts were compared using Wilcoxon signed rank test since data were paired and non-normally distributed. All comparisons were carried out using GraphPad Prism (GraphPad Software version 7.0, San Diego, CA, USA). *p*-values less than 0.05 were considered statistically significant.

## 3. Results

Regarding animals included in the study, no significant difference was present between the two treatment groups regarding weight at entry (T = −0.33, df = 263; *p* = 0.7393). However, young bulls from the pheromone group were older than young bulls in the control group (T = −2.1647, df = 263, *p* = 0.031) (see Table 1). No significant difference was observed between the two treatment groups regarding number of cow–calf farms of origin per batch (T = 0.29214, df = 21, *p* = 0.773).

### 3.1. Clinical Signs

Clinical data were obtained for 237 young bulls (137 in the pheromone group and 100 in the control group) on D8 and 230 young bulls (139 in the pheromone group and 91 in the control group) on D30. On D8, 15% (36/237) of the young bulls were affected by BRD. The diagnosis was established when the animal displayed: (i) nasal or ocular discharge or cough; and (ii) increase in respiratory rate or depression. The morbidity rate was 19% (26/137) in the pheromone group and 1% (10/100) in the control group. On D30, 8% (19/230) of the young bulls were sick with a morbidity rate of 4% (6/139) in the pheromone group and 14% (13/91) in the control group.

The results of the logistic regression model (Table 4) show that the interaction between exposure to pheromone and day of clinical exam was significantly (*p* = 0.001) associated with disease, showing that more clinical cases (OR = 8.20) were observed in the control group compared to the pheromone group on D30, while more clinical signs were observed in the pheromone group than in the control group on D8.

### 3.2. Activities and Behaviors

Activities and behaviors were observed for the 265 young bulls on D0, D8, and D30. The proportion of animals standing was significantly higher on D8 and D30 than on D0 (*p* < 0.001) for all pens. The statistical model did not show any effect of the treatment group on the proportion of animals standing idling, eating food at the feeding trough, or ruminating. Over the entire period of observation, animals from the pheromone group moved significantly more than animals from the control group (*p* < 0.05).

**Figure 1:**
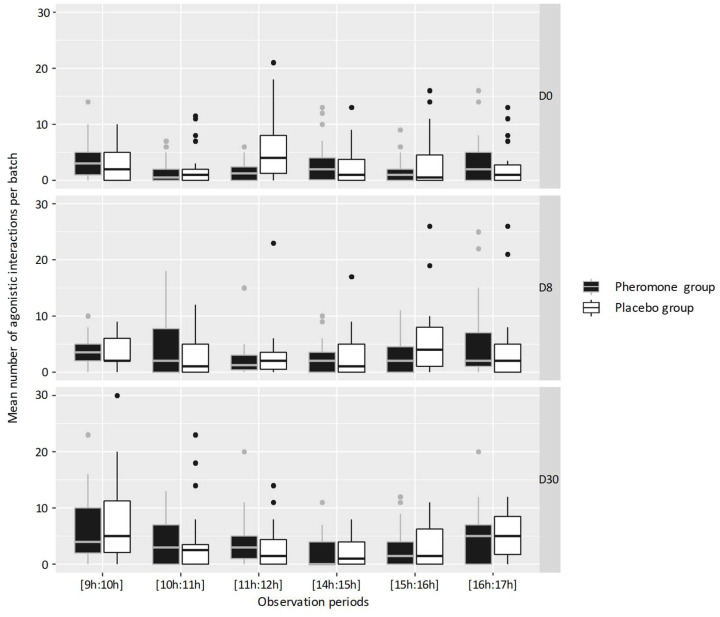
Activity and behavioral observations.

No effect of day of observation (D0, D8, or D30) or treatment on the number of agonistic interactions was demonstrated by the statistical model used. Analysis of the results shows that the number of agonistic interactions increases depending on the number of animals in a batch, regardless of the batch considered (*p* < 0.05) (Figure 1). Too few non-agonistic interactions and stereotypies were observed to allow meaningful statistical analysis.

### 3.3. Average Daily Gain

The ADG for the entire fattening period was observed for 164 young bulls. The results of the mixed linear regression model show that exposure to pheromone was not significantly associated with ADG (Table 5).

### 3.4. Assessment of Immune Transcript Expression by Quantitative Polymerase Chain Reaction

**Figure 2:**
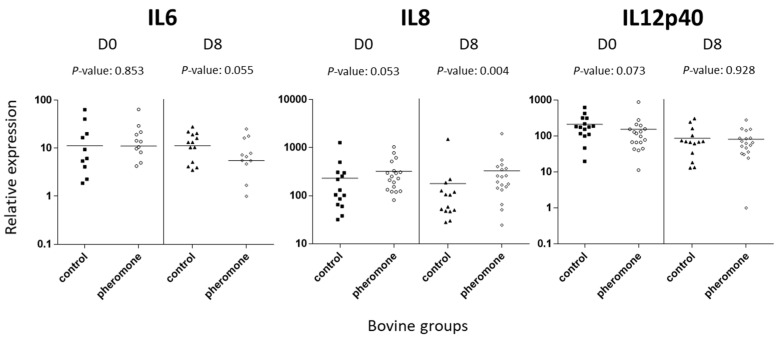
Blood IL6, IL8, and IL12p40 transcripts are differentially expressed between treated and untreated young bulls.

To assess the impact of the pheromone treatment on the expression of various immune transcripts, we collected blood from the young bulls, extracted RNA from the cells, and measured the expression of different transcripts. The selected transcripts were associated with the different common lymphocyte T helper responses (Th1, Th2, Th17, and T regulator) as well as with inflammation for the last ones (see Table 3). On D0 and D8, we did not observe significant differences (*p* > 0.05) for the vast majority of the assessed transcripts (Table 6). However, an increasing trend for IL12p40 transcripts in the blood of control group bulls in comparison to the blood of pheromone group bulls was identified (*p* = 0.073) (Figure 2 and Table 6). Similarly, an increasing trend for IL6 transcripts in control group was observed on D8 (*p* = 0.055) (Figure 2 and Table 6). Interestingly, for IL8, significant differences were identified on D8 with more IL8 transcripts in the pheromone group compared to control group (see Figure 1 and Table 6). On D0, the trend was similar, but the difference was not significant (*p* = 0.053).

## 4. Discussion

The fattening period in beef cattle is one of the most challenging phases within the production cycle. Indeed, young animals are exposed to multiple stresses of different natures with major consequences in terms of welfare, health, and productivity. Stress, especially when consecutive to weaning and transportation, has a major role in the onset of various respiratory infections during fattening through its deleterious effects on the immune defenses [2,5,35]. Bacterial respiratory infection is one of the main causes of antimicrobial molecule use during the metaphylaxis procedure and curative treatments [2,7,8]. The development and spreading of antimicrobial resistance makes the identification and establishment of alternative approaches to antimicrobial molecules to reduce their use necessary. Among the potential alternatives, bovine appeasing pheromone is an attractive one. As stated above, several previous reports have shown a benefit when appeasing pheromones were used in dairy and beef cattle production [22,24,25,26]. In our study, carried out in fattening units from western France using Charolais young bulls, we observed that significantly more clinical cases were detected on day 30 (D30) in the control group than in the group of bulls which received appeasing pheromone, while the opposite was observed earlier on D8. This observation is both similar and different from what was previously reported by Colombo and collaborators [26]. Indeed, in their study, incidence of BRD was greater (*p* < 0.05) in Angus steers that received appeasing pheromone than in the control group during Days 6–10 and 19–23, although overall BRD incidence did not differ between treatments. Moreover, a higher proportion (*p* = 0.04) of the appeasing pheromone group diagnosed with BRD required one antimicrobial treatment to retrieve health compared to control group [26]. Similarly, in our study, we detected more clinical signs in the pheromone group than in the control group early, on D8. Conversely, on D30, there were fewer BRD-associated clinical signs in the pheromone group than in the control group. Several differences between studies could be identified. For instance, the timing of pheromone administration and the measured parameters were different. In our study, we determined the presence or absence of BRD without any monitoring of pathogens, while Colombo and collaborators monitored the cumulative incidence of BRD clinical signs during a 45-day feedlot-receiving period of beef steers administered an appeasing pheromone at feedlot entry and measured the serum concentration of antibodies against various pathogens [26]. Regarding activities and behavior, we did not observe any differences between groups. Similarly, the mixed linear regression model showed that exposure to pheromone was not significantly associated with final ADG. In other studies [25,26], the authors identified positive impacts of appeasing pheromone over body weight gain; however, the benefit was not sustained throughout the 45-day experiment in *Bos indicus*-influenced beef cattle [25]. Moreover, in these studies, the authors measured intermediate ADG, while we assessed final ADG.

In the last part of our study, we assessed the whole blood expression of various transcripts associated with the immune response. The analysis of blood has been demonstrated to be a valuable approach to assess various health aspects in bovine [36,37]. In our study, we selected a limited number of target genes encompassing Th1, Th2, Th17, and Treg cytokines, as well as the associated transcription factors and chemokines. Most of the time, no significant differences were observed between control and pheromone groups of bulls on both Day 0 and Day 8 (after pheromone administration). However, for IL8 transcripts, we observed a clear difference between the pheromone group and control group on Day 8 with a higher expression of IL8 transcripts in the pheromone group than in the control group. Meanwhile, more respiratory clinical signs were identified in the pheromone group than in the control group. IL8 or CXCL8 is a well-known cytokine which was first purified and molecularly cloned as a neutrophil chemotactic factor from lipopolysaccharide-stimulated human mononuclear cell supernatants [38,39,40]. Bovine IL8 has similar roles to its human counterpart and can massively attract neutrophils to the sites of inflammation consecutive to tissue alterations induced by respiratory pathogens [41,42,43]. A positive effect of increased IL8 transcripts might be suggested in our conditions, especially since we observed fewer clinical cases of BRD in the pheromone group than in the control group on Day 30. A higher amount of IL8 in the pheromone group than in the control group could have attracted more neutrophils to the sites of inflammation and thus could have promoted a quicker resolution of respiratory infections in the pheromone group. This hypothesis is supported by observations from multiple reports, where IL8 protective effects against respiratory infections—particularly bacterial infections as it is less clear for viral infections—were identified [40,44,45]. Moreover, administration of recombinant bovine IL8 was shown to also have positive effects on uterine health and milk production [46]. Thus, monitoring of blood IL8 transcripts in young bulls could potentially bring information regarding the onset of clinical respiratory signs associated with infectious diseases. Was the increased expression of IL8 transcripts altered by pheromone treatment or was it just a direct and simple consequence of tissue damages caused by respiratory pathogens infecting bulls during the fattening period? That question cannot be answered here. However, IL8 definitely participates in the resolution of bacterial respiratory infections and superinfections through its action on neutrophils. Furthermore, it would be important to assess the IL8 protein concentration and respiratory infections over time in bovine treated with appeasing pheromones. Indeed, we cannot totally exclude discrepancies between transcript and protein expressions. Additionally, there was a clear trend for a lower expression of the IL6 transcripts in the pheromone group than in the control group. This could account for a low inflammatory status in pheromone group but further research is required to confirm this hypothesis. It would have been interesting to follow the concentrations of glucocorticosteroids and acute phase proteins such as fibrinogen in the blood compartment, as was carried out in some reports [25,26,46]. Moreover, some measures of IL8 transcript expression and protein production in the control and pheromone groups on D30 would be informative. We did not do this in the current study, which would be a valuable addition to a future experiment as an attempt to link immune transcript expressions to stress and inflammatory global status.

In our study, appeasing pheromones showed, after 30 days, a slight beneficial impact on bovine health, which is in line with previous studies [22,24,25,26] also reporting an interest in using these molecules in cattle to improve animal health.

## 5. Conclusions

In the current study, we showed that appeasing pheromone may have an impact on the bovine respiratory disease complex in the context of the fattening period. Our results confirm the potential of appeasing pheromones as a complementary approach in the management of young bulls in fattening units. However, further studies are warranted to determine the extent of the beneficial effects of appeasing pheromones observed on Day 30 and precisely decipher the mechanisms underlying these effects.

## Figures and Tables

**Table 1:** Distribution of young bulls (YB) by batches and study Group (“pheromone” or “control”).

		YB ^1^	Age	Body Weight	Pheromone Group					Control Group				
					Batches	YB	YB per Batches	Age	Body Weight	Batches	YB	YB per Batches	Age	Body Weight
Fattening unit	1	60	338 (±51)	391 (±27)	3	36	12	343 (±55)	391 (±31)	2	24	12	335 (±48)	390 (±21)
	2	90	324 (±43)	377 (±22)	4	60	15	319 (±45)	377 (±25)	2	30	15	328 (±37)	378 (±14)
	3	60	338 (±47)	371 (±25)	2	30	15	339 (±52)	382 (±26)	2	30	15	337 (±39)	361 (±18)
	4	55	261 (±49)	323 (±20)	5	33	3 × 6 1 × 5 1 × 10	246 (±52)	316 (±15)	3	22	1 × 10 2 × 6	284 (±37)	336 (±17)
Total		265	320 (±54)	370 (±32)	14	159		312 (±58)	372 (±36)	9	106		325 (±45)	368 (±26)

^1^ YB, Young Bull. The age is in days and the body weight in kilograms.

**Table 2:** List of activities, behaviors, and stereotypies observed and their respective description.

Activity, Behavior and Stereotypy			Description
Activity		Ruminating	Chewing regurgitated boluses of feed
		Eating feed at the feeding trough	Eating and masticating at the feeding trough
		Lying	Lying down in any resting position
		Standing idling	Standing
Behavior	Agonistic	Fighting	Engaging in headbutts
		Escaping	One young bull escaping from another hostile young bull
		Threatening	One young bull has a hostile behavior but no contact is made
	Non-agonistic	Chin-resting	One young bull places its chin on another young bull
		Grooming	One young bull licks another
		Sniffing	Sniffing another young bull
		Social rubbing	Rubbing another young bull
Stereotypy		Licking	Licking any equipment
		Rubbing	Rubbing repetitively own body against any equipment
		Tongue-rolling	Twisting and twirling the tongue, either inside or outside the open mouth, for at least 5 s

**Table 3:** Primer abbreviations, full names, sequences, amplicon sizes (bp), annealing temperatures (°C), and accession number or reference.

Primer Abbreviation and Full Names		Primer Sequences: Sense (S) and Anti-Sense (AS)	Amplicon Sizes (bp)	Annealing Temperatures (°C)	Accession Number or References
REFERENCE GENES	ACTB	S: ACGGGCAGGTCATCACCATC	166	67	28
	Beta actin	AS: AGCACCGTGTTGGCGTAGAG			
	GADPH	S: GGCATCGTGGAGGGACTTATG	186	62	28
	Glyceraldehyde-3-phosphate dehydrogenase	AS: GCCAGTGAGCTTCCCGTTGAG			
CYTOKINES	IL12p40	S: CACCAGCAGCTTCTTCATCA	105	60	33
	Interleukin 12 subunit p40	AS: TACTCCCAGCTGACCTCCAC			
	IL4	S: GCCACACGTGCTTGAACAAA	63	60	30
	Interleukin 4	AS: TCTCAACAGCTTGGCAAGCA			
	IL6	S: TAAGCGCATGGTCGACAAAA	150	60	32
	Interleukin 6	AS: TTGAACCCAGATTGGAAGCAT			
	IL8 (CXCL8)	S: AGAACTTCGATGCCAATGCAT	150	60	NM_173925
	Interleukin 8	AS: GGGTTTAGGCAGACCTCGTTT			
	IL17A	S: TCGTTAACCGGAGCACAAACT	120	60	32
	Interleukin 17A	AS: TGGCCTCCCAGATCACAGA			
	IL10	S: AGAACCACGGGCCTGACA	121	60	32
	Interleukin 10	AS: ACCGCCTTGCTCTTGTTTTC			
	TGFß	S: TGCTTCAGCTCCACAGAAAAGA	116	60	32
	Transforming growth factor ß	AS: AGGCAGAAATTGGCGTGGT			
	IFNɣ	S: TTGAATGGCAGCTCTGAGAAAC	150	60	32
	Interferon ɣ	AS: TCTCTTCCGCTTTCTGAGGTTAGA			
CHEMOKINES	CXCL6	S: GAGAGCTGCGTTGTGTGTGT	107	60	29
	Chemokine (C-X-C motif) ligand 6	AS: ACTTCCACCTTGGAGCACTG			
	CCL20	S: TTCGACTGCTGTCTCCGATA	172	62	28
	Chemokine (C-C motif) ligand 20	AS: GCACAACTTGTTTCACCCACT			
TRANSCRIPTION FACTORS	FOXP3	S: TGGTGCAATCTCTGGAGCAA	116	60	30
	Forkhead box P3	AS: GTCAGATGATGCCGCAGATG			
	GATA-3	S: CCAGACCAGAAACCGAAAAA	234	62	31
	Trans-acting T-cell-specific transcription factor GATA-3	AS: ACCATACTGGAAGGGTGGTG			
	RORɣ (RORC gene)	S: ACAGCCCTCGTCCTCATCAATGCC	145	60	30
	RAR-related orphan receptor gamma	AS: TGGGTGGCAGCTTTGCCAGGATA			
	TBX21	S: CGAGGACTATATACTGCCGC	133	61	31
		AS: CAAGACCACGTCCACATACA			

**Table 4:** Association between the exposure to pheromone and BRD diagnosis (diseased or healthy). The data were collected from 265 young bulls.

Variables and Levels		Odds Ratio			*p*-Value
		Estimate	95% Confidence Interval		
			Lower Bound	Upper BOUND	
Exposure to pheromone	Control	Reference			
	Pheromone	0.48	−0.20	1.10	0.84
Day of clinical examination	D8	Reference			
	D30	0.19	−0.07	0.45	0.08
Pheromone × Day of clinical examination		8.20	2.32	31.90	0.001

**Table 5:** Associations among exposure to pheromone, pen-size, and average daily gain (kg/d) of young bulls for the entire fattening period. The data were collected from 164 young bulls.

Variables and Levels		Mean ADG ^1^			*p*-Value
		Estimate	95% Confidence Interval		
			Lower Bound	Upper Bound	
Intercept		1.498			
Exposure to pheromone	Control	1.48	1.33	1.67	0.98
	Pheromone	1.48	1.33	1.67	

^1^ ADG (average daily gain) (kg/d) was calculated for the entire fattening period.

**Table 6:** Statistical comparisons between mRNA relative expressions in control and pheromone groups on Day 0 (D0) and D8. Levels of expression in controls are shown in the second column (high, amplification around 17–26 cycle quantification (*Cq*); moderate, 26–31 *Cq*; low, >31 *Cq*). *p*-values are presented in other columns. As the data were not paired and non-normally distributed, group means were compared using the Wilcoxon signed rank test (exact). ** *p* < 0.010. In bold, significant *p*-values and *p*-values nearly significant; ns, not significant; SEM, standard error of the mean.

Messenger RNAs	Levels of Expression (Controls)	D0			D8		
		mRNA Relative Expressions ± SEM		Pheromone vs. Control	mRNA Relative Expressions ± SEM		Pheromone vs. Control
		Control (*N* = 15)	Pheromone (*N* = 18)		Control (*N* = 15)	Pheromone (*N* = 18)	
CCL20	low	13.87 ± 4.13	20.72 ± 4.84	ns	12.38 ± 1.79	18.24 ± 3.26	ns
CXCL6	moderate	5.56 ± 1.64	14.77 ± 6.74	ns	5.91 ± 1.32	8.84 ± 2.18	ns
FOXP3	moderate	6.3 ± 0.9	5.85 ± 0.77	ns	4.86 ± 0.71	4.39 ± 0.71	ns
GATA-3	high	6.32 ± 0.92	8.15 ± 1.26	ns	4.72 ± 0.51	4.7 ± 0.62	ns
IFNɣ	low	18.44 ± 4.49	45.86 ± 16.27	ns	17.93 ± 3.92	37.83 ± 11.35	ns
IL10	moderate	4.15 ± 0.70	4.57 ± 1.11	ns	2.68 ± 0.51	2.76 ± 0.25	ns
IL12 p40	moderate	213.23 ± 40.59	155.05 ± 47.51	0.073	87.49 ± 22.68	82.28 ± 15.69	ns
IL17A	low	22.06 ± 10.57	34.94 ± 15.77	ns	57.53 ± 30.78	43.67 ± 14.51	ns
IL4	low	6.28 ± 4.00	0.81 ± 0.45	ns	1.13 ± 0.71	3.34 ± 2.13	ns
IL6	low	11.32 ± 4.68	11.15 ± 3.88	ns	11.33 ± 2.21	5.55 ± 1.78	0.055
IL8	moderate	231.45 ± 82.35	321.85 ± 62.56	0.053	178.25 ± 95.58	330.61 ± 104.61	0.004 **
RORɣ	moderate	6.25 ± 0.86	5.32 ± 0.46	ns	4.86 ± 0.64	5.03 ± 0.64	ns
TBX21	moderate	8.51 ± 1.47	9.33 ± 1.66	ns	4.78 ± 0.74	3.8 ± 0.60	ns
TGFß	high	3.36 ± 0.38	3.12 ± 0.32	ns	2.78 ± 0.37	3.32 ± 0.18	ns

## Data Availability

No new data were created or analyzed in this study. Data sharing is not applicable to this article.
